# Periodontal Disease Prevalence Among Diabetic Patients: A Cross-Sectional Study at a Regional Hospital in Uganda

**DOI:** 10.7759/cureus.113284

**Published:** 2026-07-24

**Authors:** Mwesigwa Amon, Evas Nimusiima, Wilfred Arubaku, Godfrey Kwizera, Isaac Okundua, Edgar Mulogo

**Affiliations:** 1 Dentistry, Brighter Smiles Dental Services, Mbarara, UGA; 2 Dentistry, Mbarara Regional Referral Hospital, Mbarara, UGA; 3 Community Health, Mbarara University of Science and Technology, Mbarara, UGA; 4 Dental Surgery, Mbarara University of Science and Technology, Mbarara, UGA

**Keywords:** diabetes, factors associated, periodontal disease (pd), periodontitis, prevalence

## Abstract

Background: The prevalence and associated factors of periodontal diseases remain scantily established in Uganda, especially in rural southwestern Uganda. Therefore, this cross-sectional study determined the prevalence and associated factors of periodontal disease among patients attending a diabetic clinic in a referral hospital in rural southwestern Uganda.

Methods: This was a hospital-based cross-sectional study conducted among 336 patients with diabetes attending a diabetic clinic at Mbarara Regional Referral Hospital (MRRH). A consecutive sampling technique was employed to enroll study participants. Bivariate and multivariate logistic regressions were employed, and variables with a p-value < 0.05 were declared statistically significant. The prevalence of periodontal disease was determined using the World Health Organization (WHO) Oral Health Assessment form for adults and categorized as gingivitis, mild periodontitis, and severe periodontitis. Community Periodontal Index (CPI) scores were determined and presented as percentages.

Results: A high prevalence of periodontal disease was recorded at 92.3%. About half of the participants (50.9%) had severe periodontitis, and only 7.7% of the participants had normal periodontitis. Being female, attaining tertiary education, consuming alcohol, visiting a dental clinic twice a year, and brushing teeth twice a day reduced the odds of developing periodontal disease.

Conclusion: The findings of this study underscore the need for integrated oral health services within diabetic clinics, with particular emphasis on oral health education and periodontal screening, especially for male patients and those with lower educational attainment.

## Introduction

Periodontal disease remains one of the most reported oral health conditions associated with diabetes and other systemic diseases, such as HIV infection and cardiovascular diseases, among others globally [1,2]. Current research studies have shown a steady increase in the prevalence and severity of periodontal disease among diabetic compared to non-diabetic patients [3,4]. Studies have continued to document a bidirectional relationship between periodontal disease and diabetes. Evidence shows that diabetes increases the prevalence, extent, and severity of periodontitis, and periodontitis negatively affects glycemic control, thus worsening the symptoms of diabetes [5,6].

In a review paper exploring this bidirectional relationship, it was revealed that the risk of developing periodontitis was two to three times higher in people with diabetes compared to individuals without. In the same paper, it was also found that treatment of periodontitis among people with diabetes resulted in improved glycemic control, with HbA1c reductions of 3-4 mmol/mol (0.3-0.4%) within three to four months after treatment [7]. On the other hand, a meta-analysis published in 2023 established that diabetes augmented the likelihood of developing or progressing into periodontitis by 86% [8]. Even with such a well-documented relationship between periodontal disease and diabetes, the prevalence of periodontitis remains scanty in Uganda. Only one research study attempted to determine the prevalence of periodontal disease among diabetes mellitus patients in Uganda. In this study, a prevalence of 85% of periodontal disease among diabetes mellitus patients was reported [9].

Data from epidemiological studies show that periodontal disease has been associated with increasing age, male gender, a low level of education, frequency of tooth brushing, frequency of dental clinic visits, poor oral hygiene, smoking, and presence of comorbidities [10,11]. However, very few studies focus on periodontal disease risk factors among diabetic patients. In Uganda, these associations remain unknown. Only a low level of education was found to be statistically significant for periodontal disease among diabetics after adjustment [9]. Therefore, this study determined the prevalence and associated factors of periodontal disease among patients attending a diabetic clinic in a referral hospital in rural southwestern Uganda.

## Materials and methods

Study design and setting

This was a cross-sectional study conducted at the diabetic clinic of Mbarara Regional Referral Hospital (MRRH), rural Southwestern Uganda, from July to December 2023. MRRH serves the whole of southwestern Uganda but also receives patients from the central region of Uganda and neighboring East African countries, especially Rwanda, Tanzania, and the Democratic Republic of Congo. This facility is the largest outpatient diabetic clinic in southwestern Uganda, and it has a patient census of approximately 1,500 patients annually. The facility also enrolls about four new cases of diabetes per week.

Study participants and sampling

The study participants were selected following this inclusion criteria: aged above 18 years: diagnosed with diabetes at least in the past one year and above; not having any other systemic diseases; not having any history of diabetic complications, such as neuropathy, nephropathy, retinopathy, etc.; not using drugs, such as phenytoin, nifedipine, etc.; not undergoning any periodontal treatment since last one year; and willingness to participate in the study.

The study excluded participants who had diabetic emergencies, such as diabetic ketoacidosis or hypoglycemia. The study had a sample size of 336 respondents. This was determined using the Kish & Leslie formula of 1965, commonly applied for prevalence and cross-sectional studies. We used a prevalence of 32.3%, which was obtained from the MRRH clinical report of 2022. We used a 95% confidence level, 5% margin of error, and 10% non-response rate. A consecutive sampling method was used to select the participants at the MRRH clinic. Every respondent who met the inclusion criteria was enrolled until the sample size was obtained.

All respondents underwent clinical examination to assess for periodontal disease, and an interviewer-administered questionnaire was used to obtain demographic data and assess for associated factors.

Each day of data collection, the research team checked the diabetes register to identify patients who were present that day and had never been recruited in this study. Eligible participants were given information regarding the nature of the study, and a written informed consent was obtained in a local language (Runyankore) from all enrolled participants by a trained research assistant. 

Ethical consideration

Ethical clearance was sought from Mbarara University of Science and Technology (MUST) - Research Ethics Committee (MUST-2023-829), and MRRH administrative permission was sought from the Hospital Director. Informed consent was obtained from all participants by signing or inserting a thumbprint on the informed consent form before they were recruited into the study. Participants enrolled were free to voluntarily withdraw from the study at any time. Confidentiality was ensured through unique identifiers rather than names, and participants were examined and interviewed in a separate room with only the research team. Collected research data were kept under lock and key, only accessible by the study team with authorization from the study Principal Investigator.

Data collection tools

An interviewer-administered questionnaire, as shown in Appendix 1, was developed after a review of relevant literature from previous studies. The questionnaire consisted of three sections: A, B, and C. Closed-ended questions were used for all sections. Section A consisted of items about social demographic characteristics: age, sex, level of education, income level, and marital status. Section B consisted of items measuring the prevalence of periodontal disease using the World Health Organization (WHO) Oral Health Assessment form for adults [12]. Individual clinical assessments were carried out by trained dental surgeons who were this study's research assistants. Section C of the questionnaire consisted of items measuring factors associated with periodontal disease. These included diabetes type, presence of comorbidities, glycemic control, frequency of tooth brushing, frequency of dental visits, smoking, and alcohol use.

A two-day training for research assistants was conducted, followed by a pilot study on the research tools to establish their reliability in capturing the required information before the actual data collection. The pilot study was conducted among 20 volunteers who met this study's inclusion criteria at a different diabetic clinic in another region. Two dental surgeons (denoted with alphabets A and B) from the research team examined the volunteer diabetic patients in the sequence below, and the scores were recorded.

A examined the first batch of 10 diabetic patients, B examined the second batch of 10 diabetic patients, A re-examined the batch of diabetic patients that B had examined earlier, and, finally, B re-examined the diabetic patients that A had examined at first. The scores were recorded independently. Then, the results were cross-matched among the dental surgeons, and from all the batches, the reason for forming batches among the diabetic patients was not to overburden them.

The examiner consistency was assessed using the kappa statistic, and this resulted in 0.924 with p-value <0.001, indicating that there was a statistically significant and almost perfect level of agreement between examiners A and B in their caries assessments. This suggested a very high degree of consistency between the two examiners.

Throughout the whole procedure, clinical assessments were conducted using a mouth mirror and a calibrated periodontal probe to measure pocket depth, which enabled us to determine the Community Periodontal Index (CPI). The CPI values were recorded as follows: score 0 - normal, score 1 - bleeding on probing, score 2 - presence of calculus with plaques seen on probing, score 3 - pockets 4-5 mm (mild periodontitis), and score 4 - pockets ≥ 6 mm. Probing was done on the buccal, palatal, mesial, and distal surfaces of each tooth. We excluded the third molars in each quadrant.

Being diabetic was measured as fasting blood sugar of >7.1 mmol/L, random of >11.1 mmol/L, and hemoglobin A1c (HbA1c) of >6.5 mmol/L. Glycemic control was measured as controlled blood sugars (HbA1c: 4-6.5 mmol/L) and uncontrolled blood sugars (HbA1c: >6.5 mmol/L).

Data analysis

Data were entered into REDCap software (version 6.5; Vanderbilt University, Nashville, TN), cleaned, and exported to STATA (version 17; StataCorp LLC, College Station, TX) for analysis. The prevalence of periodontal disease was categorized according to participant CPI value scores. Participants with CPI values of 0 were recorded as normal, participants with CPI values of 1, 2, and 3 were recorded as having gingivitis, participants with CPI values of 4-5 were recorded to have mild periodontitis, and participants with CPI values of 6 and above were recorded to have severe periodontitis.

Bivariate and multivariate logistic regression were done to assess the association between the independent factors and periodontal disease. Variables that were significant p < 0.2 at 95% confidence interval at bivariate analysis were included in the multivariate model. Further variables that were not significant from the bivariate analysis but had biological and social plausibility in relation to the outcome were included in the model. Significance was set at p-value <= 0.05. Adjusted odds ratios and 95% confidence intervals were reported.

## Results

Participant's demographic characteristics

The study recruited 336 participants with a population median age of 56 (20, 91). Close to three-quarters (67.0%, 225/336) of the participants were females, and almost the same number of participants (67.3%, 226/336) had families either officially married or cohabiting. Less than half (45.5%, 153/336) of the participants had attained an elementary class. Slightly more than half (56.8%, 193/336) of participants were earning less than USD 2 per day, and less than one third (21.4%, 72/336) of participants had uncontrolled sugar levels, as shown in Table 1.

**Table 1:** Participant sociodemographic characteristics IQR: interquartile range; USD: United States dollar

Variable	Frequency, N=336 (%)
Age (years)	
Median (IQR)	56 (20, 91)
Sex	
Male	111 (33)
Female	225 (67)
Marital Status	
Single	13 (3.9)
Married/cohabiting	226 (67.3)
Divorced	18 (5.4)
Widowed	79 (23.5)
Level of Education	
No formal education	85 (25.3)
Elementary school	153 (45.5)
High school	68 (20.2)
Tertiary	30 (8.9)
Income Level	
=< 2 USD per day	193 (57.4)
>2 USD per day	143 (42.6)
Glycemic Control	
Controlled sugar levels	264 (78.6)
Uncontrolled sugar levels	72 (21.4)
Periodontal Disease	
Normal	26 (7.7)
Gingivitis	123 (36.6)
Mild periodontitis	16 (4.8)
Sever periodontitis	171 (50.9)

Prevalence of periodontal disease among diabetic patients

**Figure 1:**
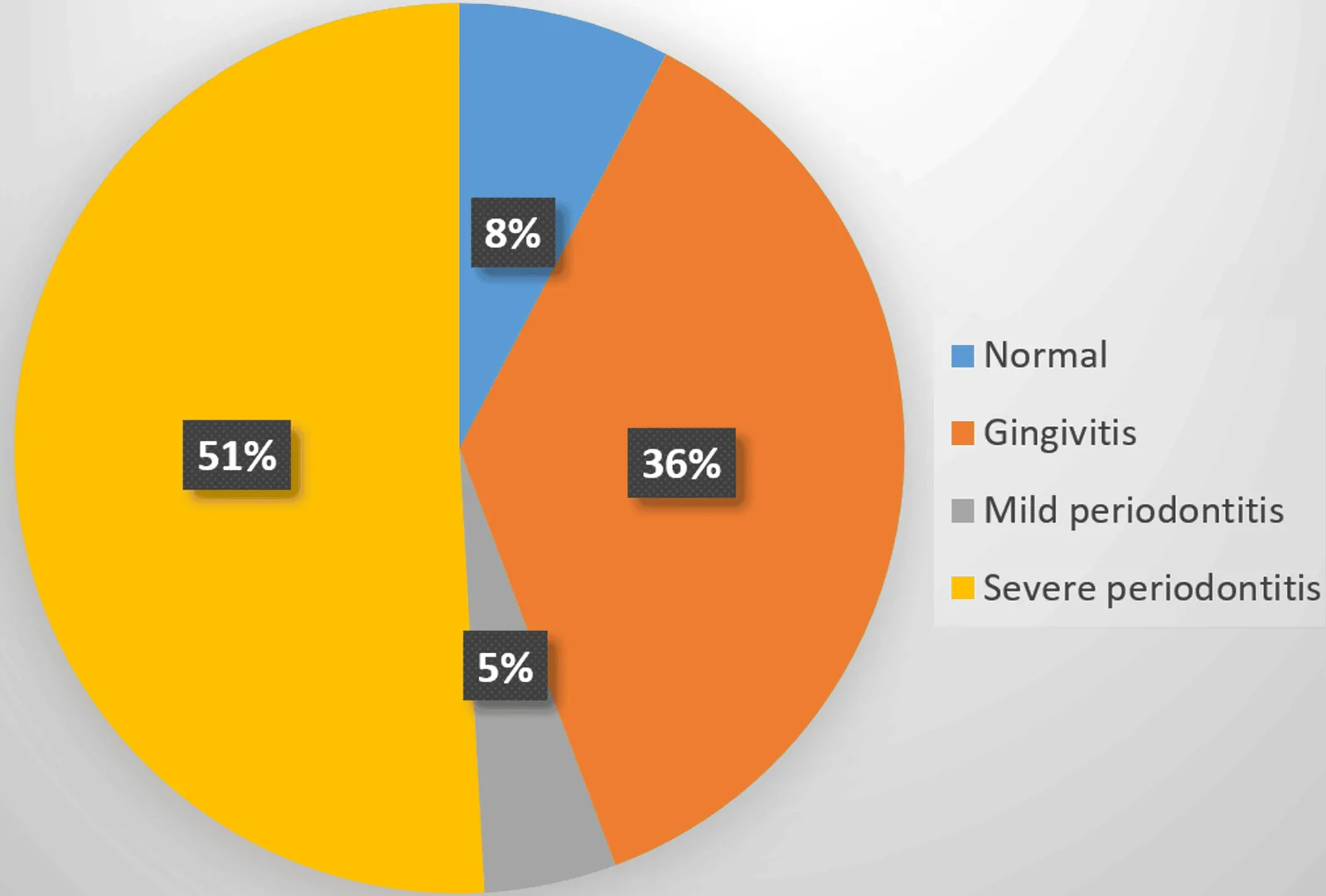
Prevalence of periodontal disease among diabetic patients

Overall, the prevalence of periodontal disease was recorded at 92.3% (310/336). Almost a third (36.6%, 123/336) of the participants had gingivitis, a small proportion (4.8%, 16/336) had mild periodontitis, and half of the participants (50.9%, 171/336 ) had severe periodontitis. On the other hand, only (7.7%, 26/336) were normal without any form of periodontal disease, as shown in Figure 1.

Factors associated with periodontal disease among diabetic patients

Tables 2-3 show the factors associated with periodontal disease at bivariate and multivariate analysis, respectively. Results from this study showed that female participants had reduced odds of developing periodontal disease at bivariate analysis (OR: 0.244, 95%CI: 0.072-0.831, p = 0.024), and similar findings were statistically significant at the multivariate level (AOR: 0.170, 95%CI: 0.041-0.702, p = 0.014). Participants with tertiary education had much lower odds of developing periodontal disease at bivariate analysis (OR: 0.146, 95%CI: 0.034-0.629, p = 0.010). This finding was statistically significant at multivariate analysis (AOR: 0.151, 95%CI: 0.028-0.805, p = 0.027). Participants who consumed alcohol had slightly lower odds of developing periodontal disease compared to their counterparts at the bivariate level (OR: 0.230, 95%CI: 0.083-0.637, p = 0.005). Similar findings were statistically significant after adjustment (AOR: 0.109, 95%CI: 0.030-0.393, p = 0.001). Participants who visited a dental clinic twice a year had lower odds of developing periodontal disease as compared to their counterparts at the bivariate level (OR: 0.267, 95%CI: 0.761-0.935, p = 0.039). This finding was not statistically significant after adjustment (AOR: 0.296, 95%CI: 0.067-1.301, p = 0.107). Increasing age, smoking, and presence of comorbidities were associated with increased odds of developing periodontal disease at bivariate analysis (OR: 1.771, 95%CI: 0.766-4.094, p = 0.181; OR: 4.598, 95%CI: 0.608-34.740, p = 0.139; OR: 1.75, 95%CI: 0.783-3.910, p = 0.172, respectively); however, they were not statistically significant after adjustment (OR: 1.235, 95%CI: 0.461-3.304, p = 0.674; OR: 3.613, 95%CI: 0.380-34.321, p = 0.263; OR: 1.449, 95%CI: 0.628-4.094, p = 0.428, respectively).

**Table 2:** Factors associated with periodontal disease among diabetic patients at bivariate analysis * statistical significance at bivariate analysis at P-value <0.2. COR: crude odds ratio; USD: United States dollar

Characteristics	Bivariate Analysis				
	Periodontal Disease	No Periodontal Disease	COR	95%CI	P-value
Age (years)					
Below 56	153 (90.0)	17 (10.0)	1		
Above 56	157 (94.6)	9 (5.4)	1.938	0.838-4.481	0.122*
Sex					
Male	108 (97.3)	3(2.7)	1		
Female	202 (89.8)	23(10.2)	0.244	0.072-0.831	0.024*
Marital Status					
Single	8 (61.5)	5 (38.5)	1		
Married/cohabiting	213 (94.2)	13 (5.8)	10.240	2.934-35.736	0.0001
Divorced	17 (94.4)	1 (5.6)	10.625	1.059-106.573	0.045
Widowed	72 (91.1)	7 (8.9)	6.429	1.649-25.056	0.007
Income Level					
=<2 USD per day	180 (93.3)	13 (6.7)	1		
>2 USD per day	130 (90.9)	13 (9.1)	0.681	0.301-1.543	0.358
Level of Education					
No formal education	82 (96.5)	3 (3.5)	1		
Elementary school	144 (94.1)	9 (5.9)	0.585	0.154-2.223	0.432
High school	60 (88.2)	8 (11.8)	0.274	0.699-1.078	0.064*
Tertiary	24 (80.0)	6 (20.0)	0.146	0.034-0.629	0.010*
Diabetes Type					
Type 1	91 (91.9)	8 (8.1)	1		
Type2	218 (92.4)	18 (7.6)	1.060	0.445-2.525	0.896
Presence of Comorbidities					
No	124 (89.9)	14 (10.1)	1		
Yes	186 (93.9)	12 (6.1)	1.75	0.783-3.910	0.172*
Frequency of Tooth Brushing					
No brushing	40 (95.2)	2 (4.8)	1		
Once a day	185 (94.9)	10 (5.1)	0.949	0.200-4.501	0.947
Twice a day	85 (85.9)	14 (14.1)	0.311	0.067-1.437	0.135*
Smoke					
No	262 (91.3)	25 (8.7)	1		
Yes	48 (98.0)	1 (2.0)	4.598	0.608-34.740	0.139*
Frequency of Dental Visits					
Never	165 (93.8)	11 (6.2)	1		
Once a year	125 (92.6)	10 (7.4)	0.833	0.343-2.024	0.687
Twice a year	16 (80.0)	4 (20.0)	0.267	0.761-0.935	0.039*
More than thrice a year	4 (80.0)	1 (20.0)	0.267	0.027-2.593	0.255
Alcohol Use					
No	290 (93.5)	20 (6.5)	1		
Yes	20 (76.9)	6 (23.1)	0.230	0.083-0.637	0.005*

**Table 3:** Factors associated with periodontal disease among diabetic patients at multivariate analysis * Statistical significance at multivariate analysis at P-value <0.05. AOR: adjusted odds ratio

Periodontal disease	AOR	95%CI	P value
Age (years)			
Belo 56	1		
Above 56	1.235	0.461-3.304	0.674
Sex			
Male	1		
Female	0.169	0.410-0.702	0.014*
Education Level			
No formal education	1		
Elementary school	0.582	0.137-2.44	0.460
High school	0.262	0.059-1.169	0.079
Tertiary	0.151	0.028-0.805	0.027*
Presence of Comorbidities			
No	1		
Yes	1.449	0.579-3.623	0.428
Smoking			
No	1		
Yes	3.613	0.380-34.321	0.263
Alcohol Use			
No	1		
Yes	0.109	0.300-0.393	0.001*
Frequency of Dental Visits			
Never	1		
Once a year	0.981	0.379-2.534	0.968
Twice a year	0.296	0.067-1.301	0.107
More than twice a year	0.208	0.017-2.481	0.215

## Discussion

This study aimed to assess the prevalence and associated factors of periodontal disease among diabetic patients attending an outpatient diabetic clinic at MRRH. From this study's findings, it was found that the prevalence of periodontal disease was very high among diabetic patients. This finding is consistent with previous studies that have reported a high prevalence ranging 80-90% [9,13]. Most studies have attributed this high prevalence to the bidirectional relationship that exists between periodontal disease and diabetes. Chronic hyperglycemia promotes the formation and accumulation of advanced glycation end products (AGEs), which bind to their receptors (RAGE) on immune and endothelial cells, stimulating the release of pro-inflammatory cytokines, including tumor necrosis factor-alpha (TNF-α), interleukin-1β (IL-1β), and interleukin-6 (IL-6) [14]. This persistent inflammatory response impairs neutrophil function, disrupts collagen metabolism, delays wound healing, and enhances alveolar bone resorption, thereby increasing susceptibility to periodontal tissue destruction [15]. In turn, periodontal inflammation contributes to systemic inflammation, which increases insulin resistance and compromises glycemic control, reinforcing the bidirectional relationship between the two conditions [6,16,17]. Notably, in our study, 21% of the participants had uncontrolled blood sugar. A similar study conducted in Uganda reported a high prevalence of periodontal disease above 85% with a HbA1c mean score of 8.8 mmol/L, an indicator of the existence of this bidirectional relationship between periodontal diseases and poor glycemic control among study participants [9].

This study found that females are less likely to develop periodontal disease than their male counterparts. These results are consistent with other literature that highlights men as being at a higher risk of periodontal disease as compared to females [9,18,19]. As compared to males, females tend to exhibit better oral hygiene practices and are more proactive in visiting dentists [20]. Moreover, females with poor oral hygiene practices are still less prone to developing periodontal disease than their male counterparts [9]. Notably, females are less exposed to other risk factors of periodontal disease than men. For example, females are less likely to be current or former smokers than males, thus less likely to develop periodontal disease [9,21,22]. This necessitates gender-tailored interventions educating males about proper oral hygiene practices and proper health-seeking behaviors.

In this study, having a tertiary education was protective against periodontal disease, which concurs with other literature that associates low levels of education with a higher prevalence of periodontal disease [23]. This is also in line with a study that was conducted at a referral hospital in Uganda that found an association between lower levels of education and periodontal disease [9]. Educational level is a social determinant of health and a measure of one's economic status, and its effect on general well-being has been well documented [24]. Individuals with a higher level of education are more likely to be richer and therefore able to afford periodontal treatments, which are, in most cases, expensive, reducing their likelihood of suffering from the disease [9]. Additionally, higher education promotes health literacy, thus better health practices such as proper oral hygiene and social habits, which are essential in lowering periodontal disease risks [25,26]. Health educating masses in the lower socio-economic class about better health practices could bridge the literacy gap and, in turn, reduce the incidence of periodontal disease.

Surprisingly, alcohol consumption was slightly associated with lower odds of periodontal disease (AOR: 0.139, 95%CI: 0.042-0.466). This finding contradicts the majority of published literature, which reports a positive association between alcohol intake and periodontitis [27,28]. Individuals with high alcohol intake show changes in subgingival microbial composition and higher proportions of pathogens responsible for periodontal disease [29]. However, another study about alcohol and periodontal pockets showed no association between the two [30]. Another cohort study in Japan showed no association between alcohol consumption and the incidence of periodontal disease [28]. The very small number of participants who reported alcohol consumption (n = 26) and the lack of data on frequency, quantity, and type of alcoholic beverage limit the interpretation of this finding in this study. It is possible that individuals with advanced periodontal disease reduced or stopped alcohol intake due to oral pain or medical advice (reverse causation), or that residual confounding by unmeasured socioeconomic or behavioral factors exists. Focused studies exploring these drinking habits in the study population could possibly clarify the observed relationship in this study.

This study is one of the few studies in Uganda that systematically examined periodontal status using the WHO CPI. It was performed by qualified dental surgeons rather than relying on self-report data from participants. The consecutive recruitment of 336 diabetic patients from a large regional referral hospital's diabetic clinic enhances the representativeness of diabetic patients seeking care in rural southwestern Uganda.

However, some limitations should be acknowledged. This was a clinic-based study that employed consecutive sampling with a fairly tight inclusion criterion. These factors could have introduced selection bias, affecting the representativeness of the study sample and the generalizability of the study findings. The very high prevalence of periodontal disease (92.3%) may have reduced the power to detect weaker associations. Also, alcohol consumption was recorded only as a binary variable without details on quantity, frequency, or type, limiting the interpretation of the unexpected protective association observed. Notably, although we adjusted for several confounders, residual confounding by unmeasured factors like detailed oral hygiene practices, use of traditional oral hygiene tools, or genetic predisposition cannot be excluded. These considerations should be considered in future studies about this topic. Despite the limitations, this study still provides valuable insights into factors associated with periodontal disease among diabetic patients.

## Conclusions

This study demonstrates a very high prevalence of periodontal disease among diabetic patients attending a diabetic clinic at a regional referral hospital in rural southwestern Uganda, with half of the participants presenting severe periodontitis. Female sex and tertiary education were protective factors against periodontal disease. The unexpected protective slight association with reported alcohol consumption is likely explained by measurement limitations.

These findings underscore the need for integrated oral health services within diabetic clinics in Uganda, with particular emphasis on oral health education and periodontal screening targeted at male patients and those with lower educational attainment. Future longitudinal studies with detailed exposure assessment (especially of alcohol and oral hygiene practices) are warranted to confirm these associations and evaluate the impact of periodontal treatment on glycemic control in this population.

## Appendices

Appendix 1

**Table 4:** Data collection tool - Section A

Participant ID: ____________________			
Date: ________ / ________ / __________			
Name of Research Assistant: _______________________________________________			
Section A: Sociodemographic Characteristics			
No.	Item	Responses	
1	Age	__________ years	
	Sex	☐ Male	
		☐ Female	
2	Marital status	☐ Single	
		☐ Married/cohabiting	
		☐ Divorced	
		☐ Widowed	
3	Level of education	☐ No formal education	
		☐ Elementary school	
		☐ High school	
		☐ Tertiary	
4	Level of income	☐ ≤ 2 USD per day	
		☐ > 2 USD per day	

**Table 5:** Data collection tool - Section B

Section B: Prevalence of Periodontal Disease		
Parameter	Score	Respondent's Score (Tick)
Respondent has no periodontal disease	0	
Respondent bleeds on probing	1	
Respondent has calculus with plaque seen or felt by probing	2	
Respondent has a pathological pocket 4-5 mm	3	
Respondent has pathological pocket 6 mm and above	4	
Respondent has only one tooth present, or no teeth are present	X	

**Table 6:** Data collection tool - Section C

Section C: Factors Associated With Periodontal Disease Among Diabetic Patients		
No.	Item	Responses
1	Age	__________________ (years)
2	Sex	☐ Male
		☐ Female
3	Glycemic control	☐ Controlled sugar levels
		☐ Uncontrolled sugar levels
4	Type of diabetes	☐ Type 1
		☐ Type 2
5	Respondent has comorbidities (e.g., HIV, hypertension, TB, etc.)	☐ Yes
		☐ No
6	Do you smoke?	☐ Yes
		☐ No
7	Do you consume alcohol?	☐ Yes
		☐ No
8	I have ever heard about dental caries	☐ Yes
		☐ No
9	Diabetes puts me at risk of dental caries	☐ Yes
		☐ No
		☐ I don't know
10	Frequency of dental visits	☐ Never
		☐ Once a year
		☐ Twice a year
		☐ More than twice a year
11	Frequency of brushing	☐ No brushing
		☐ Once a day
		☐ Twice a day
12	Have you ever been referred by a healthcare provider for a dental ...	☐ Yes
		☐ No
